# Characteristics of the Earliest Cross-Neutralizing Antibody Response to HIV-1

**DOI:** 10.1371/journal.ppat.1001251

**Published:** 2011-01-13

**Authors:** Iliyana Mikell, D. Noah Sather, Spyros A. Kalams, Marcus Altfeld, Galit Alter, Leonidas Stamatatos

**Affiliations:** 1 Seattle BioMed, Seattle, Washington, United States of America; 2 Department of Global Health, University of Washington, Seattle, Washington, United States of America; 3 Division of Infectious Diseases, Department of Medicine, Vanderbilt University School of Medicine, Nashville, Tennessee, United States of America; 4 Ragon Institute of Massachusetts General Hospital, Massachusetts Institute of Technology and Harvard University, Boston, Massachusetts, United States of America; University of Zurich, Switzerland

## Abstract

Recent cross-sectional analyses of HIV-1+ plasmas have indicated that broadly cross-reactive neutralizing antibody responses are developed by 10%–30% of HIV-1+ subjects. The timing of the initial development of such anti-viral responses is unknown. It is also unknown whether the emergence of these responses coincides with the appearance of antibody specificities to a single or multiple regions of the viral envelope glycoprotein (Env). Here we analyzed the cross-neutralizing antibody responses in longitudinal plasmas collected soon after and up to seven years after HIV-1 infection. We find that anti-HIV-1 cross-neutralizing antibody responses first become evident on average at 2.5 years and, in rare cases, as early as 1 year following infection. If cross-neutralizing antibody responses do not develop during the first 2–3 years of infection, they most likely will not do so subsequently. Our results indicate a potential link between the development of cross-neutralizing antibody responses and specific activation markers on T cells, and with plasma viremia levels. The earliest cross-neutralizing antibody response targets a limited number of Env regions, primarily the CD4-binding site and epitopes that are not present on monomeric Env, but on the virion-associated trimeric Env form. In contrast, the neutralizing activities of plasmas from subjects that did not develop cross-neutralizing antibody responses target epitopes on monomeric gp120 other than the CD4-BS. Our study provides information that is not only relevant to better understanding the interaction of the human immune system with HIV but may guide the development of effective immunization protocols. Since antibodies to complex epitopes that are present on the virion-associated envelope spike appear to be key components of earliest cross-neutralizing activities of HIV-1+ plasmas, then emphasis should be made to elicit similar antibodies by vaccination.

## Author Summary

A fraction of those infected with HIV develop broadly neutralizing antibodies (bNAbs) capable of preventing cell-infection by diverse HIV isolates; the type of antibodies we wish to elicit by vaccination. Identifying factors associated with the natural development of bNabs, and defining the timing of their emergence and their epitope specificities, will assist the development of more effective immunogens and vaccination protocols. Here we performed a neutralization screen of plasma samples collected longitudinally from HIV-1-infected subjects and determined that on average, cross-neutralizing antibody responses emerge 2–3 years, but as early as one year, following infection. A significant portion of the earliest cross-neutralizing antibody response to HIV targets epitopes that are present on the virion-associated trimeric Env spike, but not the corresponding soluble monomeric versions of that viral protein. Our study highlights the importance of eliciting by vaccination antibodies with this type of complex epitope specificities.

## Introduction

The initial antibody response to the HIV-1 viral envelope glycoprotein (Env) manifests itself within the first 2 weeks of infection and is non-neutralizing [1], [2]. Autologous neutralizing antibodies develop during the first months after infection [3], [4], [5] and recent studies indicated that approximately 10%–30% of chronically-infected HIV-1 subjects develop cross-reactive neutralizing antibody responses of significant breadth [6], [7], [8]. These latter responses are the ones an effective vaccine should elicit [9]. Several studies indicated that the breadth of plasma cross-neutralizing antibody responses is positively associated with plasma viral load [6], [7], [10], [11], [12], but very little is known about the time course of these responses. A recent study by van Gils et al, using samples collected at 2 and 4 years following infection, indicated that a greater number of infected subjects displayed cross-neutralizing activities at 4 than at 2 years [12]. However, the earliest timing of the development of such responses was not determined. Defining the timing of emergence of cross- neutralizing antibody responses following HIV-1 infection and identifying factors associated with their development, will advance our understanding of the complex interaction of HIV-1 with the immune system, will improve our understanding on how HIV-1 infection leads to immune dysfunction, and will also be useful to the development of immunization protocols that hopefully would elicit similar antibody responses.

The epitope specificities of the anti-HIV-1 cross-reactive neutralizing antibody responses in HIV-1+ plasmas collected during chronic infection are complex, with many specificities remaining undefined. Although there is general consensus that these neutralizing activities rarely target the transmembrane subunit gp41, but mostly the extracellular gp120 subunit [7], [13], [14], [15], [16], [17], there remains quite an uncertainty whether the overall cross-neutralizing activities of HIV-1+ plasmas are due to a single, a limited number of, or many different epitope specificities [7], [13], [14], [15], [16], [18], [19], [20], [21], [22]. The above studies were conducted with samples from chronically-infected subjects and very little, if anything, is known about the epitope specificities of the earliest cross-neutralizing antibody responses in HIV-1+ plasmas. Defining these epitope specificities would be informative for future immunogen design efforts.

Here we analyzed the cross-neutralizing antibody responses in longitudinal plasmas collected soon after and up to seven years after HIV-1 infection. We found that the subset of HIV-1-infected subjects that develop cross-neutralizing antibody responses do so on average within the first 2.5 years of infection, although in rare cases such responses became detectable as early as 1 year after infection. Epitope-mapping analyses indicated that the earliest cross-neutralizing antibody responses target primarily epitopes within and around the CD4-BS of gp120, or epitopes that are present on the virion-associated trimeric Env, but not on the corresponding monomeric gp120 or gp41 Env subunits. In contrast, the neutralizing activities of plasmas from subjects that did not develop cross-neutralizing antibody responses, target epitopes on monomeric gp120, other than the CD4-BS. These observations are indicative of the presence and long-term survival of B cells that recognize complex but conserved epitopes on the viral Env in those HIV-infected subjects that develop cross-neutralizing antibody responses.

## Results

### Detection of cross-reactive neutralizing responses as early as one year following HIV-1 infection

**Figure 1:**
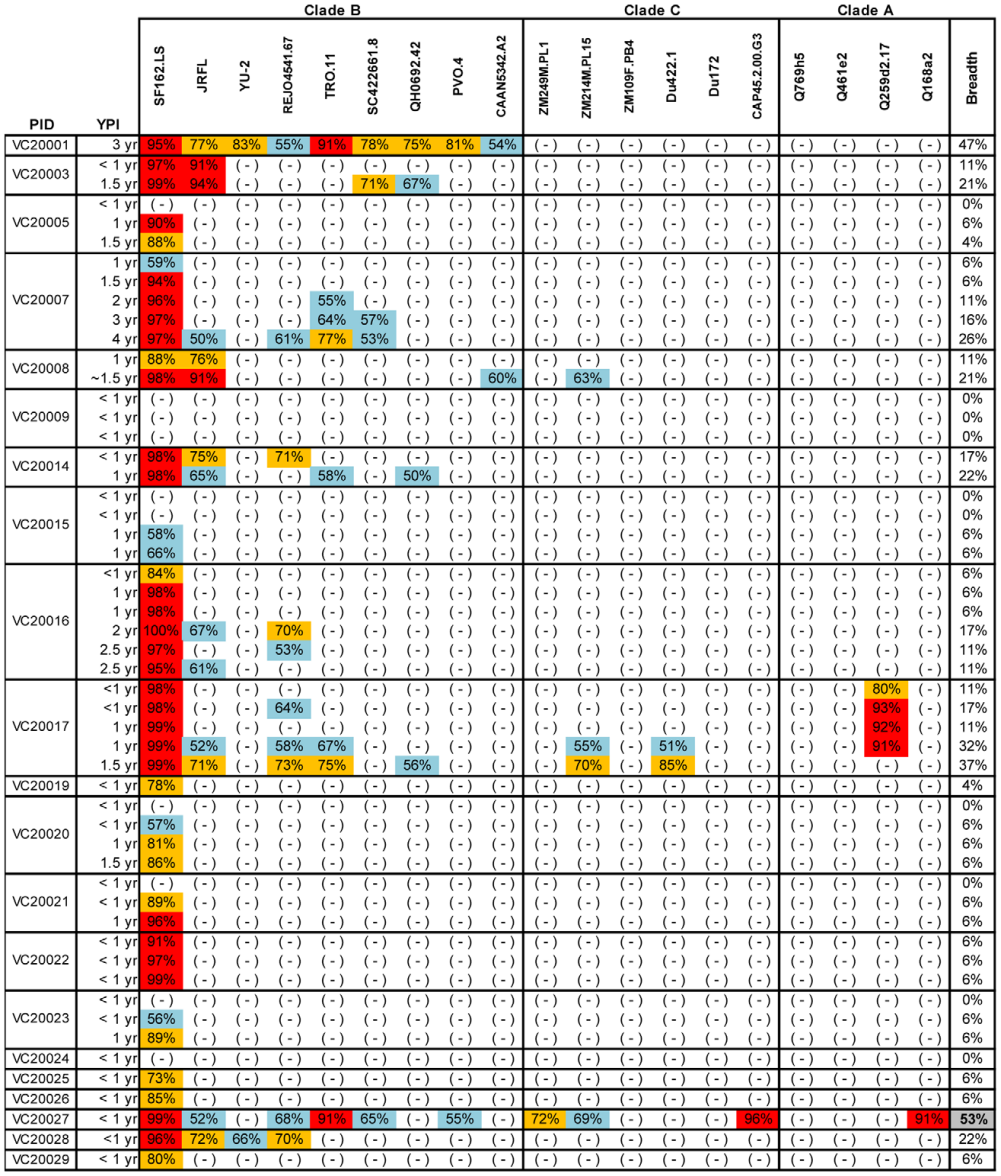
Cross-neutralizing activities in plasmas from the Vanderbilt Cohort. The cross-neutralizing activities of plasmas from the indicated subjects (PID) were evaluated against the indicated clade B, C and A viruses. The values are the plasma titers at which 50% neutralization (IC50) was recorded. For clarity this information is color-coded: (blue) IC50<1∶100; (orange) 1∶100≤IC50≥1∶250; (red) IC50>1∶250. With the exception of SF162.LS (tier 1 virus), all other viruses are tier 2 [58], [59], [61]. (-): less than 50% neutralization was recorded; YPI: years post-infection; ‘breadth’: the percent of isolates neutralized by a plasma sample, out of the total number of isolates tested, irrespective of the potency of neutralization [7]. Each experiment was performed at least two independent times.

**Figure 2:**
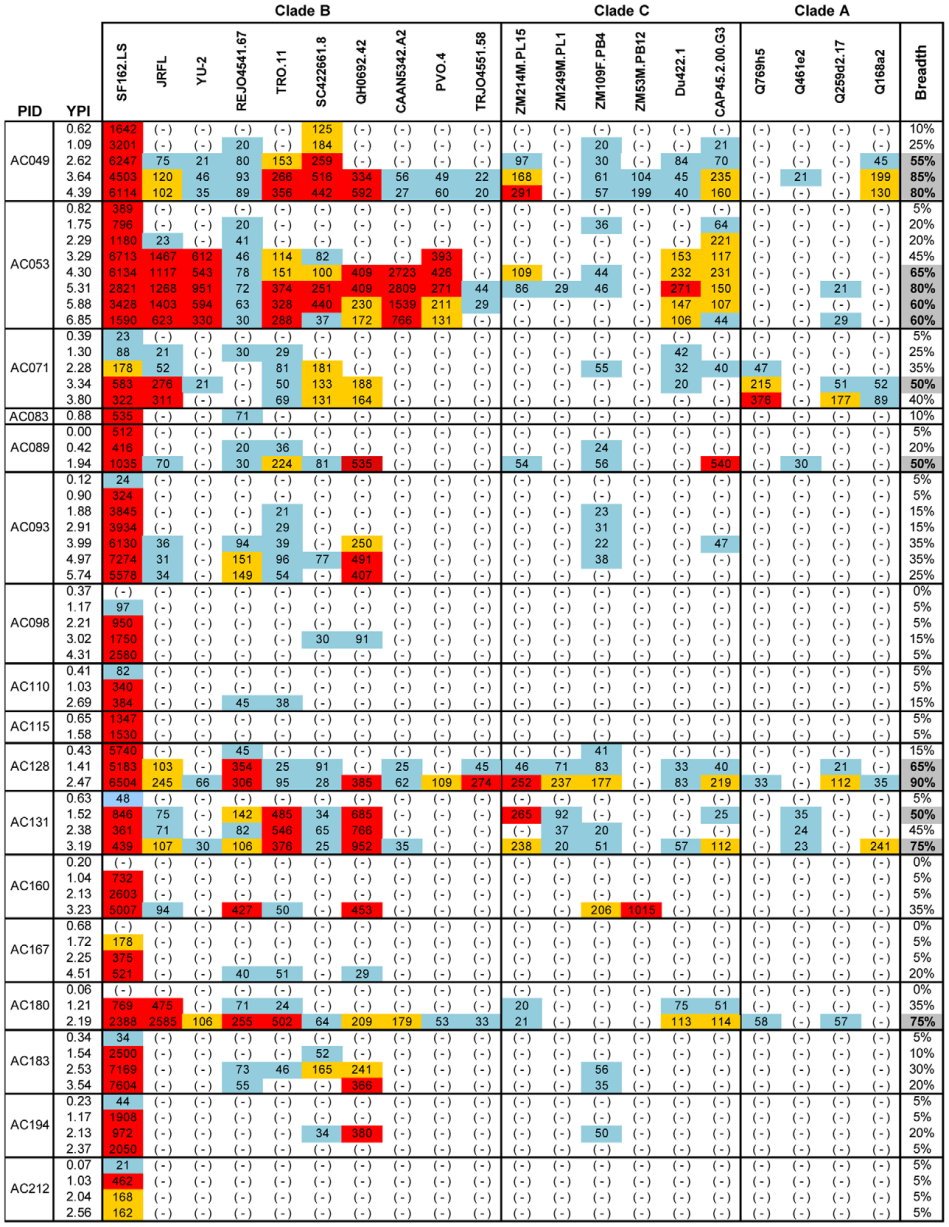
Cross-neutralizing activities in plasmas from the MGH Acute HIV infection Cohort. The cross-neutralizing activities of plasmas from the indicated subjects (PID) were evaluated against the indicated clade B, C and A viruses. The values are the plasma titers at which 50% neutralization (IC50) was recorded. For clarity this information is color-coded: (blue) IC50<1∶100; (orange) 1∶100≤IC50≥1∶250; (red) IC50>1∶250. With the exception of SF162.LS (tier 1 virus), all other viruses are tier 2 [58], [59], [61]. (-): less than 50% neutralization was recorded; YPI: years post-infection; ‘breadth’: the percent of isolates neutralized by a plasma sample, out of the total number of isolates tested, irrespective of the potency of neutralization [7]. Each experiment was performed at least two independent times.

To define the earliest period following HIV-1 infection when cross-neutralizing antibody responses appear in plasma we determined the neutralizing activities of plasmas collected within a few months and up to several years post HIV-1 infection from anti-retroviral naïve subjects infected with clade B viruses, against 20 heterologous clade A, B and C primary isolates (Figures 1 and 2). Plasma samples from two independent cohorts were examined. The samples from the Vanderbilt cohort (VC) were, for the most part, collected within the first year of infection (Figure 1). The breadth of cross-neutralizing activity (i.e., the percentage of viruses neutralized by any given plasma out of the total number of viruses the plasma was tested against) was minimal (less than 50%), in agreement with previous observations [2], [5]. In most cases, these ‘early’ plasmas efficiently neutralized the ‘easy-to-neutralize’ primary SF162.LS virus, but not other primary viruses examined here. In the few cases where neutralizing activity against viruses other than SF162 was observed, the potency of neutralization was for the most part very weak and the neutralizing activities targeted clade B viruses. In two cases (subjects VC20017 and VC20027) the ‘early’ plasmas also neutralized a few non-clade B viruses. Plasma VC20027 collected within the first year of infection neutralized 6/9 clade B, 3/6 clade C and 1/4 clade A viruses. Plasma sample collected from subject VC20017 during the first year of infection neutralized 4/9 clade B viruses, the clade A virus Q259d2.17, and the clade C viruses ZM214M and Du422.1. These observations indicate that cross-neutralizing antibody responses begin to emerge during the first year of HIV-1 infection, but that such responses are weak in potency and narrow in breadth; rarely targeting viruses from clades other than the one the patient is infected with.

**Figure 3:**
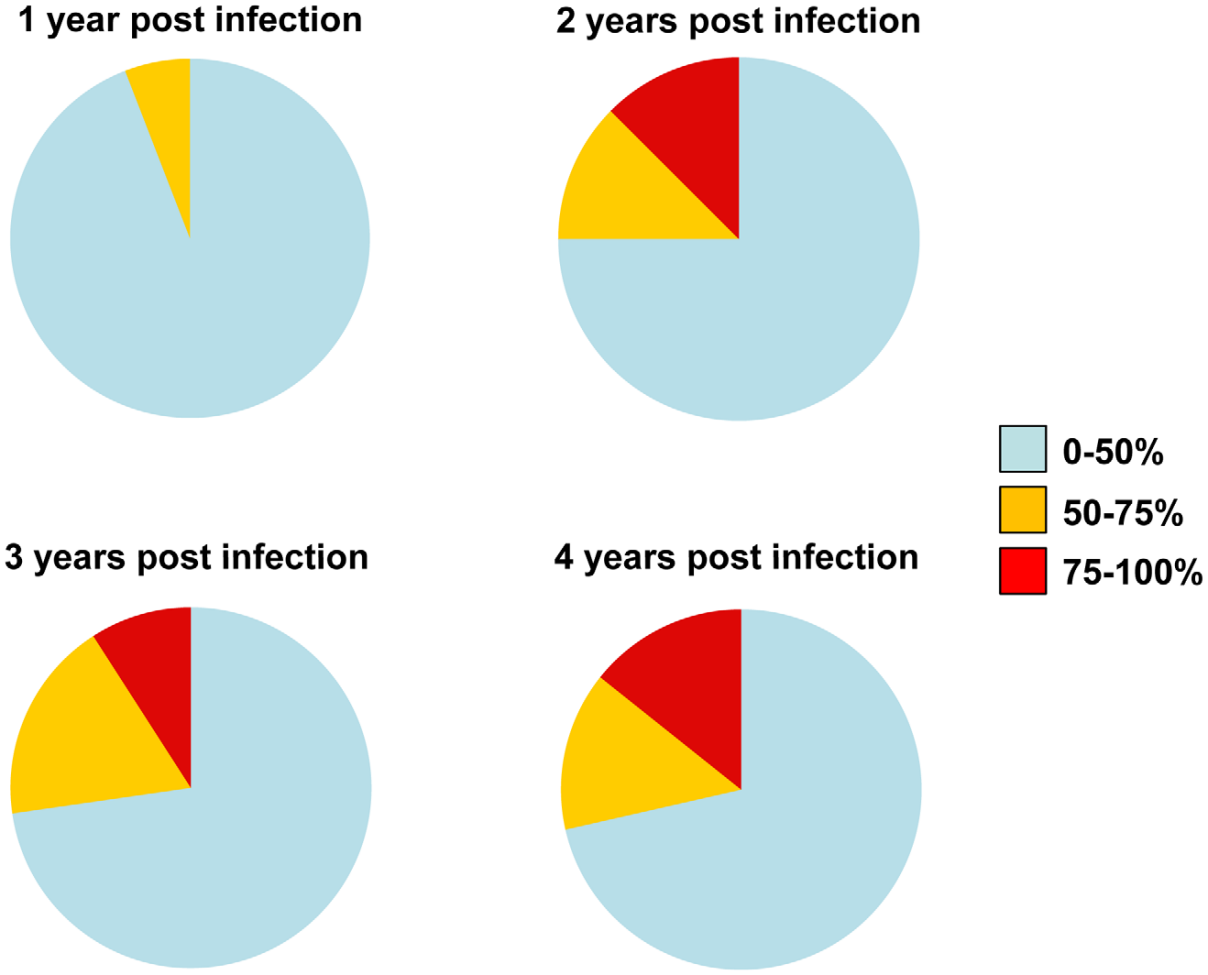
Evolution of the potency and breadth of cross-neutralizing antibody responses. The pie charts represent the evolution of breadth and potency of serum neutralizing activities in the MGH Acute HIV Infection Cohort. Subjects who developed cross-neutralizing activities within the first 2 years of infection, but who were not followed longitudinally post that period, were not included in our calculations in the subsequent years. Samples from 17 subjects were available during the 1^st^ year of infection, samples from 16 during the 2^nd^ year, 11 during the 3^rd^, and 7 during the 4^th^ year.

In the case of the MGH Acute HIV Infection Cohort (AC), plasma samples were collected longitudinally within a few months after infection and up to approximately 7 years post infection, with an average follow-up of 3.31 years. Here, too, samples collected during the first year of infection did not display broad cross-neutralizing activities (Figure 2). In only one case (subject AC128), a plasma sample collected approximately 1.4 years after infection neutralized 65% of the heterologous viruses tested (7/10 clade B, 5/6 clade C and 1/4 clade A). A plasma sample collected a year later from the same subject neutralized 90% of the viruses tested with greater potency: an indication of a continuous evolution and increase in the breadth of the cross-neutralizing antibody responses during the first 2 years of infection in this subject. Overall, plasma samples from 7/17 subjects (41%) (AC049, AC053, AC071, AC089, AC128, AC131, and AC180) displayed cross-neutralizing activities against 50% of the isolates tested against at some point during the period of observation. Samples from 5/17 subjects (29%) (AC049, AC053, AC128, AC131, and AC180) displayed broad cross-neutralizing activities against at least 75% of the viruses tested at some point during the period of observation. This percentage is in agreement with numerous previous reports on the frequencies of broad cross-neutralizing activities in sera collected during chronic HIV-1 infection [6], [7], [8]. In these 5 cases, a gradual increase in the breadth of cross-neutralizing activities was recorded over time, even though plasmas collected longitudinally from individual subjects did not always neutralize the same isolates, nor with the same potency. Such changes in potency by samples collected over time from individual subjects could be due to changes in the number of epitopes recognized by the circulating antibodies (i.e., the relative proportions of NAbs with diverse epitope specificities change over time), and/or due to changes in the plasma concentrations of antibodies with epitope specificities that do not change over time. Collectively, the above results indicate that the mean time it took for the breadth of cross-neutralizing activities to reach 50% was 2.13 years and the mean time to reach 75% was 3.08 years. Although the percentage of subjects developing cross-neutralizing antibody responses increased during the first 3 years of infection, that percentage did not further increase in year 4 (Figure 3). Clearly, additional longitudinal analysis is required to determine whether cross-neutralizing antibody responses increase past that time.

### Factors associated with the emergence of cross-neutralizing antibody responses

**Figure 4:**
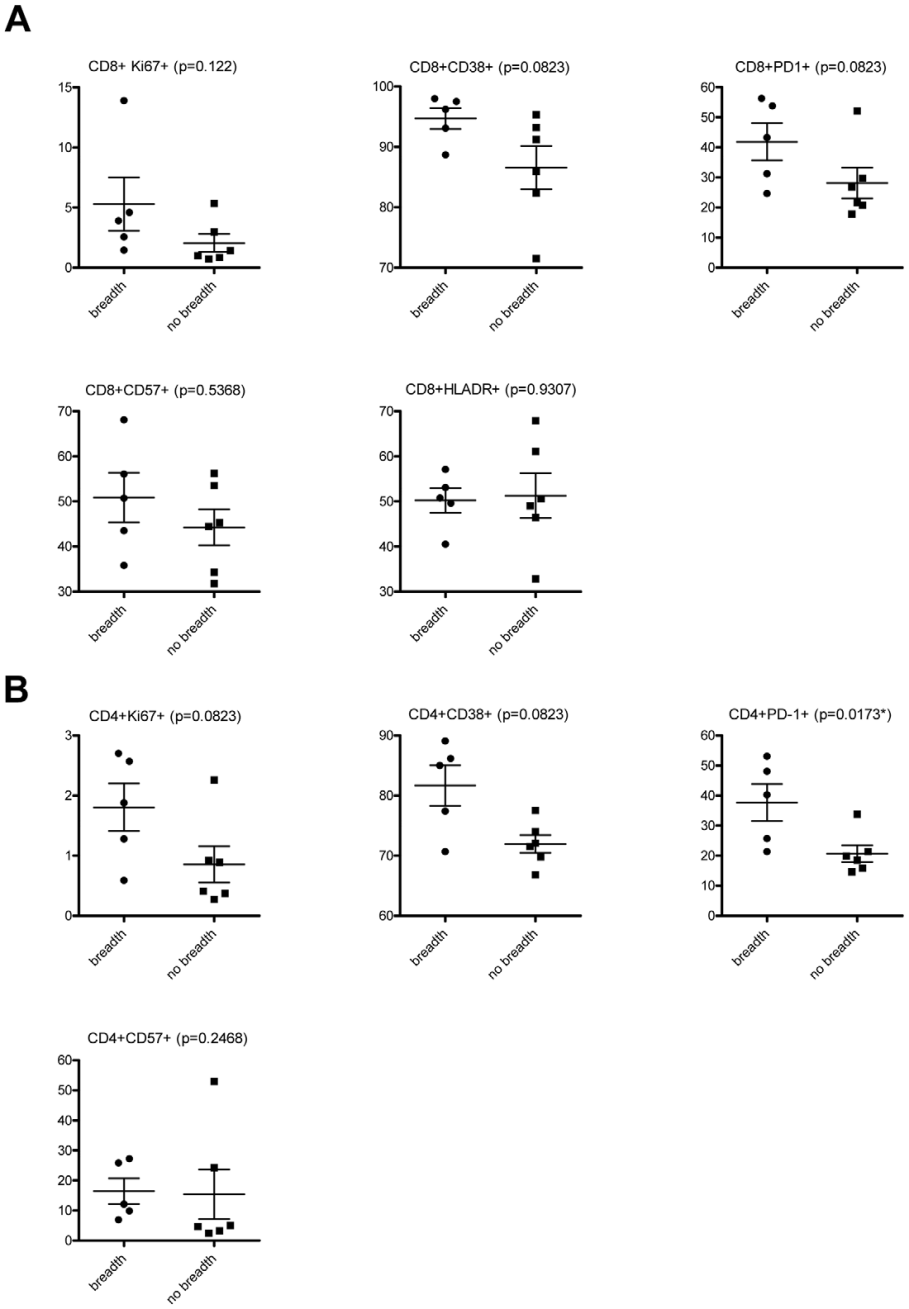
Immune activation markers and cross-neutralizing antibody responses. The frequencies of (A) CD8+, and (B) CD4+ T cells expressing the indicated markers in subjects who developed cross-neutralizing antibodies (at least 75% breadth) (AC049, AC053, AC128, AC131 and AC180) and those who did not (no breadth) (AC093, AC110, AC167, AC183, AC194, AC212) are shown. These frequencies were determined at the earliest time point when cross-neutralizing antibody responses were evident: for AC049 at 2.62 year post-infection (ypi), for AC053 at 3.29 ypi, for AC128 at 1.41 ypi, for AC131 at 1.52 ypi, and for AC180 at 2.19 ypi. Similar time points of infection were used for those subjects who did not develop cross-neutralizing antibody responses.

In chronic HIV-1 infection the breadth of serum cross-neutralizing antibody activities positively correlates with the levels of plasma viremia [6], [7], [11], [12]. Here, we recorded a positive correlation (p = 0.0026; R = 0.3615) between the breadth of the earliest cross-neutralizing antibody responses in HIV+ plasmas and the levels of plasma viremia. Because of the association between plasma viremia and immune activation during early HIV infection [23], we examined potential associations between the development of serum cross-neutralizing activities and markers of immune activation and exhaustion (Figure 4). Specifically, we compared the percent of CD4+ and CD8+ T cells expressing Ki67, CD57, CD38, PD1, and HLADR in subjects that developed cross-neutralizing antibody responses and those who did not. The immune activation status of subjects who developed broad cross-neutralizing antibody responses (at least 75% breadth at some point during the period of observation) was determined at the earliest time point when cross-neutralizing antibody responses were evident: for AC049 at 2.62 year post-infection (ypi), for AC053 at 3.29 ypi, for AC128 at 1.41 ypi, for AC131 at 1.52 ypi, and for AC180 at 2.19 ypi. Similar time points of infection were used for those subjects who did not develop cross-neutralizing antibody responses (AC093, AC110, AC167, AC183, AC194, and AC212).

A trend towards higher percentages of CD8+ T cells expressing Ki67 (p:0.122), CD38 (p:0.0823), and PD1 (p: 0.0823) was recorded in subjects with breadth. It is likely that, because the number of subjects who developed cross-neutralizing antibody responses is small, these differences did not reach statistical significance. A similar trend towards higher expression of Ki67 (p:0.0823) and CD38 (p:0.0823) was recorded in the case of CD4+ T lymphocytes. A statistically significant difference (P:0.0173) was, however, recorded in the percent of CD4+ PD1+ T cells between those subjects that developed cross-neutralizing antibody responses and those who did not. In addition, we performed correlation analysis between the degree of breadth and the frequencies of T cells expressing the various activation markers. A statistically significant positive association was observed between breadth and the frequency of CD4+ PD1+ T cells (p: 0.0174, Pearson r: 0.6961), and CD4+ CD38+ T cells (p: 0.0306, Pearson r: 0.6494). Overall, these results link for the first time the state of immune activation (within approximately 2 years of infection) to the development of cross-reactive neutralizing antibody responses.

### Epitope specificities of the earliest cross-neutralizing antibody response

Taking advantage of the availability of longitudinal samples from the MGH Acute HIV Infection Cohort, we performed epitope-mapping studies to determine: (a) whether the initial cross-neutralizing antibody responses developed by subjects infected with different viruses were due to the emergence of antibodies that target one or multiple epitopes on heterologous Env, and (b) whether the initial epitope specificities of cross-reactive neutralizing antibody responses in HIV-1+ plasmas evolve over time.

#### Cross-neutralizing activities targeting the transmembrane gp41 Env subunit

The extracellular part of the transmembrane subunit gp41 is immunogenic [24], [25] and the target of the initial anti-HIV-1 antibody responses generated following infection [2]. However, the vast majority of human anti-gp41 MAbs are non-neutralizing and only a handful of anti-gp41 neutralizing MAbs have been isolated from HIV-1-infected subjects [26], [27], [28], [29]. Two of these anti-gp41 MAbs, 2F5 and 4E10, display broad cross-neutralizing activities and they recognize two distinct epitopes within MPER [26], [27]. In between the 2F5 and 4E10 epitopes lies the epitope recognized by a third anti-HIV-1 antibody, Z13, whose breadth of neutralization is much narrower than those of 2F5 and 4E10 [27], [30].

We first performed peptide competition neutralization experiments, during which the plasmas were pre-incubated with a MPER-derived peptide and then incubated with viruses (JRFL and TRO.11) (Table 1). MPER-derived peptides block the neutralizing activities of MAbs 4E10 and 2F5, and have been used to define the contribution of anti-MPER neutralizing activities in HIV+ sera [7], [13], [14], [31], [32]. Control experiments confirmed that the MPER peptide specifically competes the neutralizing activities of the anti-MPER MAbs 2F5 and 4E10, but not those of neutralizing antibodies to other regions of Env (such as the anti-gp120 MAbs b12, 2G12, P3C8 and P3E1) (data not shown). In only one subject (AC131) the MPER-derived peptide consistently reduced (by a modest 0.3–0.6 Log10) the plasma's overall neutralizing activity against both HIV-1 viruses tested, indicating that the anti-MPER antibodies in this plasma moderately contribute to its overall anti-HIV-1 cross-neutralizing potential. Interestingly, the relative contribution of anti-MPER antibodies to the anti-JRFL and -TRO.11 neutralizing activity decreased during the period of observation in that subject, although the breadth of cross-neutralizing activity increased from 45% to 75% during that period.

**Table 1:** Contribution of anti-MPER antibodies to the plasma's cross-neutralizing activities.

			Log decrease in presence of MPER^d^		IC50 of HIV2/HIV1 MPER chimeras^g^			
PID^a^	YPI^b^	Breadth^c^	JRFL	TRO.11	C1 (MPER)	C3 (2F5)	C4 (4E10)	C8 (Z13, 4E10)
**AC131**	**2.38**	**45%**	**0.48**	**0.68**	**>2560**	**43**	**119**	**>2560**
	**3.19**	**75%**	**0.39**	**0.35**	**>2560**	**24**	**157**	**>2560**
AC049	3.64	85%	(--)^e^	(--)				
	4.39	80%	(--)	nd^f^				
AC053	5.31	80%	(--)	(--)				
	5.88	60%	(--)	(--)				
AC071	3.34	50%	(--)	(--)				
	3.80	40%	(--)	nd				
AC089	1.94	50%	(--)	(--)				
AC093	4.97	35%	(--)	(--)				
AC128	2.47	90%	(--)	(--)				

a Patient ID.

b Years post infection.

c Breadth: the percentage of HIV-1 isolates neutralized by each plasma, out of the total number of isolates the plasma was tested against.

d The neutralizing activities of plasmas collected at the indicated time points following infection were evaluated against the HIV-1 viruses JRFL and TRO.11 in the absence or presence of the MPER peptide (NEQELLELDKWASLWNWFDITNWLWYIRKKK). The Log10 decrease in IC50 neutralization titers in the presence of the MPER peptide is indicated.

e No affect in the neutralizing activities of plasmas was recorded in the presence of the MPER peptide.

f Experiment not performed.

g The IC50 neutralizing titers of plasma samples collected from subject AC131, against four HIV2/HIV-1 MPER chimeric viruses are indicated. The MPER amino acid sequences of the parental strains and the chimeras are as follows: 7312A HIV-2 (QIQQEKNMYELQKLNSWDVFGNWFDLASWVKYIQYGVYIV), YU-2 MPER (NEQELLALDKWASLWNWFDITKWLWYIKI), 7312A-C1 (QIQQEKNMYELLALDKWASLWNWFDITKWLWYIKYGVYIV), 7312A-C3 (QIQQEKNMYELLALDKWASLWNWFDLASWVKYIQYGVYIV), 7312A-C4 (QIQQEKNMYELQKLNSWDVFGNWFDITKWLWYIKYGVYIV), 7312A-C8 (QIQQEKNMYELQKLNSWASLWNWFDITKWLWYIKYGVYIV). For each chimeric virus, we indicate whether it expresses the 2F5, 4E10, Z13 epitopes, or the entire HIV-1 MPER The core epitopes of 2F5 (ALDKWA), Z13 (WASLWNWFDIT) and 4E10 (NWFDIT) are underlined.

To better define the epitope(s) within the MPER region targeted by these antibodies we utilized viruses expressing chimeric HIV-2/HIV-1 Envs [13], [31], [33]. These chimeric Envs are based on the HIV-2 Env 7312A, on which the entire MPER region, or portions of it, have been replaced by those of the HIV-1 clade B YU2 Env. Since plasmas isolated from HIV-1-infected subjects rarely neutralizing HIV-2 isolates, these chimeras are useful tools to detect the presence of anti-HIV-1 MPER neutralizing antibody responses [13], [31], [33]. The MPER sequences of the chimeras used here are shown in Table 1. As expected, plasma from AC131 was very effective in neutralizing the C1 chimera, which expresses the entire HIV-1 MPER region (Table 1). This plasma also potently neutralized the C8 chimera, which expresses the domain of MPER encompassing the Z13 and 4E10 epitopes, while it did not efficiently neutralize the C3 or C4 chimeras, which express the 2F5 and 4E10 epitopes, respectively, of the HIV-1 MPER. Most likely therefore, this plasma contains neutralizing antibodies whose epitope(s) overlaps the Z13 and 4E10 epitopes. Overall, these results indicate that anti-MPER neutralizing antibodies rarely (and only modestly) contribute to the earliest cross-neutralizing antibody responses following HIV-1 infection. They are in agreement with numerous recent studies indicating that anti-MPER targeted antibodies rarely contribute to the breadth of cross-neutralizing activities of HIV-1+ sera from chronic infection [7], [13], [14], [15], [16], [17], [31], [32].

We did not investigate whether anti-gp41 antibodies that target regions other than the MPER were present and contributed to the early cross-neutralizing activities of the above plasmas. Although several monoclonal antibodies against the HR1 domain of gp41 have been shown to display anti-HIV neutralizing potentials, such antibodies are generally not broadly neutralizing [28], [34], [35].

#### Cross-neutralizing activities targeting the gp120 Env subunit

**Figure 5:**
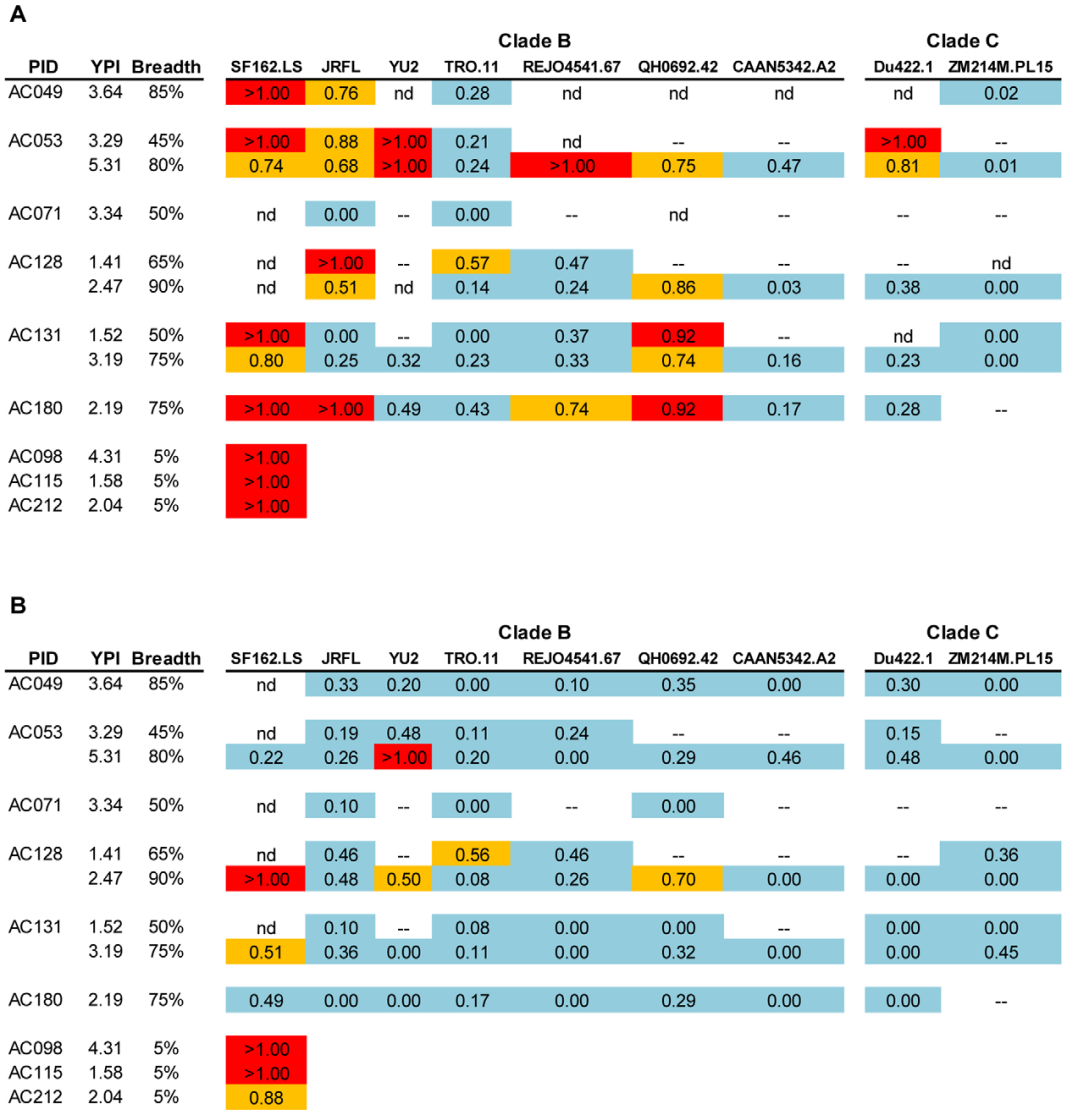
Contribution of anti-gp120 antibodies to the overall neutralizing activity of HIV+ plasmas. (A) Log10 decrease in neutralizing activity caused by the elimination of anti-gp120 antibodies, and (B) Log10 decrease in neutralizing activity of plasmas in the presence of D368R. The values are the average from 2–3 independent experiments in most cases. Light blue: no effect or less than 0.5 Log10 decrease; Yellow: decrease between 0.5 and 0.9 Log10; Red: over 0.9 Log10 decrease. >1.00: indicates that the depletion of anti-gp120 antibodies from plasma resulted in complete loss of the neutralizing activity. (--) the experiment was not performed because that particular plasma did not neutralize that particular virus; nd: experiment was not performed; YPI: years post infection.

**Figure 6:**
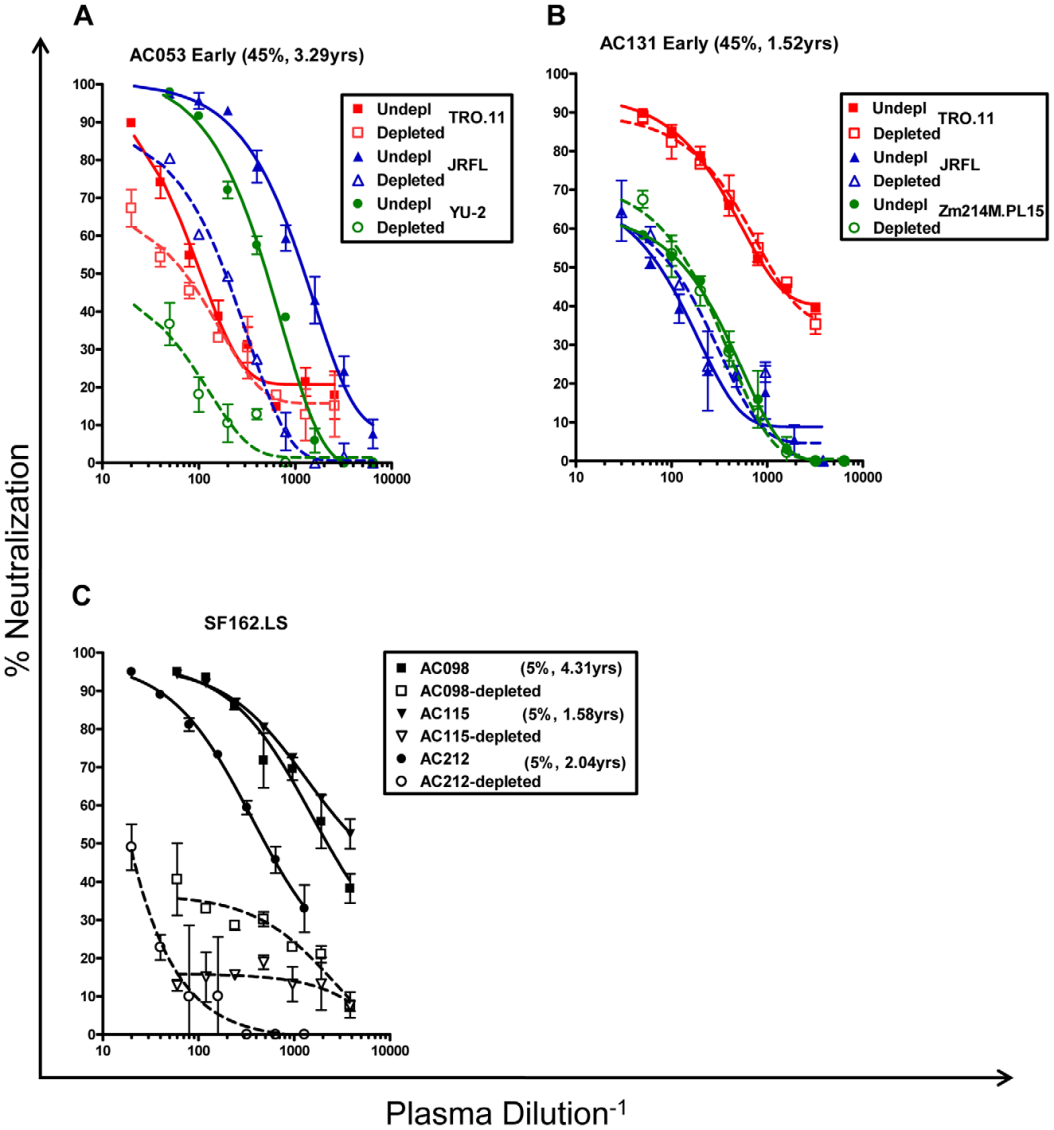
Neutralizing activities of anti-gp120 antibody-depleted plasmas. (A and B) Neutralizing activities of 2 plasmas against the indicated primary isolates prior to and following the removal of anti-gp120 antibodies, are shown. (A) Plasma AC053 against the TRO.11, JRFL, and YU2 viruses. (B) Plasma AC131 against TRO.11, JRFL, and Zm214M viruses. TRO.11 (red squares), JRFL (blue triangles), YU2 and ZM214M (green circles). Patient ID, breadth and years post infection are shown. Undepl: Undepleted plasma. Depleted: plasma depleted from anti-gp120 antibodies. Closed symbols and solid lines – undepleted plasmas; open symbols and dashed lines – gp120-depleted plasmas. (C) Plasmas from subjects AC098 (squares), AC115 (inverted triangles), and AC212 (circles) did not display significant breadth and neutralized only SF162.LS (Figure 2). Their anti-SF162.LS neutralizing activities were determined before and following depletion of the anti-gp120 antibodies. Closed symbols and solid lines: prior to depletion of anti-gp120 antibodies; open symbols and dashed lines: following the depletion of anti-gp120 antibodies. Each experiment was performed at least three independent times.

Potentially, the above results suggested that the earliest cross-neutralizing activities in HIV-1+ plasmas are primarily targeting the extracellular gp120 subunit. To address this point, we depleted the anti-gp120 antibodies from six plasmas (AC049, AC053, AC071, AC128, AC131, AC180) displaying cross-neutralizing activities, and three of the plasmas (AC098, AC115, AC212), which only neutralized SF162 (Figure S1). Although during these depletion experiments we used gp120 from one clade B virus (SF162), we verified that this treatment eliminated anti-gp120 antibodies against other gp120s, from both clade B and clade C viruses (Figure S1). Then, the neutralizing activities of non-depleted and of the corresponding gp120-antibody-depleted plasmas were compared against several clade B and C viruses (the data are summarized in Figure 5A and representative examples are shown in Figure 6). Removal of the anti-gp120 antibodies from plasmas with narrow breadth resulted in complete loss in neutralizing activity. In contrast, removal of the anti-gp120 antibodies from plasmas with breadth had a diverse effect on the neutralizing activities of plasmas, depending on the plasma / targeted virus pairing. In most cases examined, either no changes in IC50 titers were recorded or changes smaller than 0.5Log10 in IC50 were recorded. However, in specific cases the neutralizing activity of given plasma against a given virus was completely lost when the anti-gp120 antibodies were removed. That was the case of plasma AC131 and the QH0692 and SF162 viruses (but not other viruses tested); or the case of plasma AC053 and the YU2, REJO and Du422 viruses (but that was not the case for this plasma's anti-TRO.11, -CAAN or -ZM214 neutralizing activities).

It was recently reported by Walker and colleagues that a significant fraction of cross-neutralizing activities in plasmas collected during chronic HIV-1-infection are recognizing complex epitopes on the trimeric Env spike that are not efficiency presented on (or are absent from) monomeric gp120 [22]. Antibodies with epitope specificities overlapping those of MAbs PG9 and/or PG16 were defined as being partially responsible for the overall cross-neutralizing activity of some sera tested in that study. We were interested in determining whether similar PG9- and PG16-like antibody specificities are contributing to the earliest cross-neutralizing anti-HIV-1 activities.

To address this point we generated mutants of HIV-1, on which the asparagine at position 160 (within the V2 loop) was substituted by a lysine. Position 160 is critical for the binding and neutralization of MAbs PG9 and PG16 [22], [36], and recently it was suggested that the sugar molecules present on N160 may also directly participate in the binding of these two MAbs [37]. In agreement with these previous studies, we observed that when asparagine 160 was mutated into a lysine (N160K) or to an alanine (N160A), MAbs PG9 and PG16 no longer neutralized HIV-1 (TRO.11) (Figure S2). Next, the susceptibilities of the WT and mutant TRO.11 virus against plasmas were determined, once gp120-antibodies were eliminated from these plasmas (Table 2). We selected plasmas from subjects AC053, AC131 and AC181, since removal of anti-gp120 antibodies from those samples had a minimal effect on their anti-TRO.11 neutralizing activities (Figure 5A). In all cases a decrease of that plasmas' neutralizing activity was recorded in the case of the N160K mutations. However, the N160A mutation had no effect on the neutralizing activity of the same plasmas. Combined, the results indicate that the sugars at position 160 are not part of the epitope recognized by these antibodies. Potentially, they suggest that the nature of the amino acid at position 160 is very relevant to the neutralizing activity of the earliest cross-neutralizing antibody response in HIV-1+ plasmas. In summary, our results suggest that the earliest cross-neutralizing antibody response to HIV-1 includes antibodies whose epitopes on the virion-associated Env spikes are overlapping, but are not identical to those of MAbs PG9 and PG16.

**Table 2:** Percent reduction in neutralizing activity of gp120-depleted plasmas due to amino acid substitutions at position N160.

Patient ID	YPI^a^	Breadth	TRO.11 N160K	TRO.11 N160A
AC053	5.31	80%	**36%** b	(--)^c^
AC131	3.19	75%	**53%**	(--)
AC180	2.19	75%	**25%**	(--)

Gp120-depleted plasmas were tested for neutralization of TRO.11 N160 mutants.

a Years post infection.

b % neutralizing activity is the % reduction in IC50 of mutant compared to wild type.

c No difference between neutralization of wild type and mutant TRO.11.

#### Epitope-specificities on monomeric gp120

We performed epitope-mapping analysis to define the regions within monomeric gp120 that were targeted by cross-reactive neutralizing antibody responses in certain plasmas. Usually, such epitope-mapping analysis studies are performed by incubating HIV-1+ plasmas with soluble peptides derived from the variable regions of the extracellular HIV-1 Env subunit gp120 [7], [13], [14]. However, soluble peptides do not accurately represent the conformations of the variable regions of HIV-1 Env and thus do not accurately report on the contribution of antibodies that recognize conformational epitopes within the variable regions. To deal with this limitation we developed a competition neutralization assay based on a variant of monomeric gp120, termed D368R.

The D to R mutation at the conserved position 368 within the CD4-BS abrogates the binding of CD4 and of most known anti-CD4-BS MAbs to gp120 (with a few exceptions; see below), while it does not affect the binding of antibodies that target epitopes outside the CD4-BS [7], [15], [19]. Because D368R does not bind cellular CD4, it does not interfere with the ability of the virus to enter CD4+ target cells during *in vitro* neutralization assays. We confirmed that D368R competes the neutralizing activities of known MAbs that recognize epitopes outside the CD4-BS, such as P3C8 (anti-V1), P3E1 and 447-52D (anti-V3) [38], [39] and 2G12, which recognizes a conformational epitope made of mannose residues [40], [41], [42] (Figure S3A). D368R had no effect on the neutralizing activities of the anti-CD4-BS MAb b12 or of CD4-IgG2 (Figure S3B).

We next examined if the neutralizing activities of the above-described plasmas were affected (and to what extent) by the presence of D368R (the data are summarized in Figure 5B and representative examples are shown in Figure S4). The neutralizing activities of the three plasmas (AC098, AC115, and AC212) with narrow breadth were significantly reduced in the presence of D368R; an indication that the anti-SF162 neutralizing activities of these plasmas are due to antibodies that primarily target epitopes outside the CD4-BS. In contrast, the cross-neutralizing activities of plasmas with breadth were either not affected or only modestly reduced by D368R. The exception was plasma AC053, whose anti-YU2 neutralizing activity was significantly reduced by D368R. This result suggests that the anti-YU2 neutralizing activity of this plasma (but not its neutralizing activity against any other viruses tested) is due to antibodies that recognize epitopes on gp120 located outside the CD4-BS.

As expected, in the cases where depletion of the anti-gp120 antibodies had no or only minimal effect on the neutralizing activity of a plasma sample against a set of viruses, the D368R protein had a similar effect. In cases, however, where the neutralizing activity of plasma against a particular virus was significantly affected by depletion of the anti-gp120 antibodies, our results indicate that the major fraction of anti-gp120 antibodies responsible for that neutralizing activity was due to anti-CD4-BS antibodies. This is the case for example of plasmas AC049, AC053, AC128, and AC180 against JRFL, of plasma AC053 (3.29 ypi) against YU2, or plasmas AC053 (3.29 ypi), AC131 (3.19 ypi), and AC180 against the QH0692 virus.

Here we need to clarify that although the D368R mutation abrogates the binding of most known anti-CD4-BS antibodies, it does not abrogate the binding of all such antibodies. Corti et al [28] recently discussed the binding and neutralizing properties of such a MAb, HJ16. In addition, Scheid et al [16] identified a group of anti-HIV Env antibodies generated during infection, termed anti-gp120 core, whose epitopes overlap part of the CD4-BS and whose binding is not affected by the D368R mutation. Thus, anti-core antibody specificities in HIV-1+ plasmas will be ‘removed’ if the plasmas are incubated with D368R. Potentially, the fraction of the plasma neutralizing activities that was ‘eliminated’ in the presence of D368R could be due to anti-core antibodies.

#### Longitudinal evolution of anti-D368R neutralizing activities

**Figure 7:**
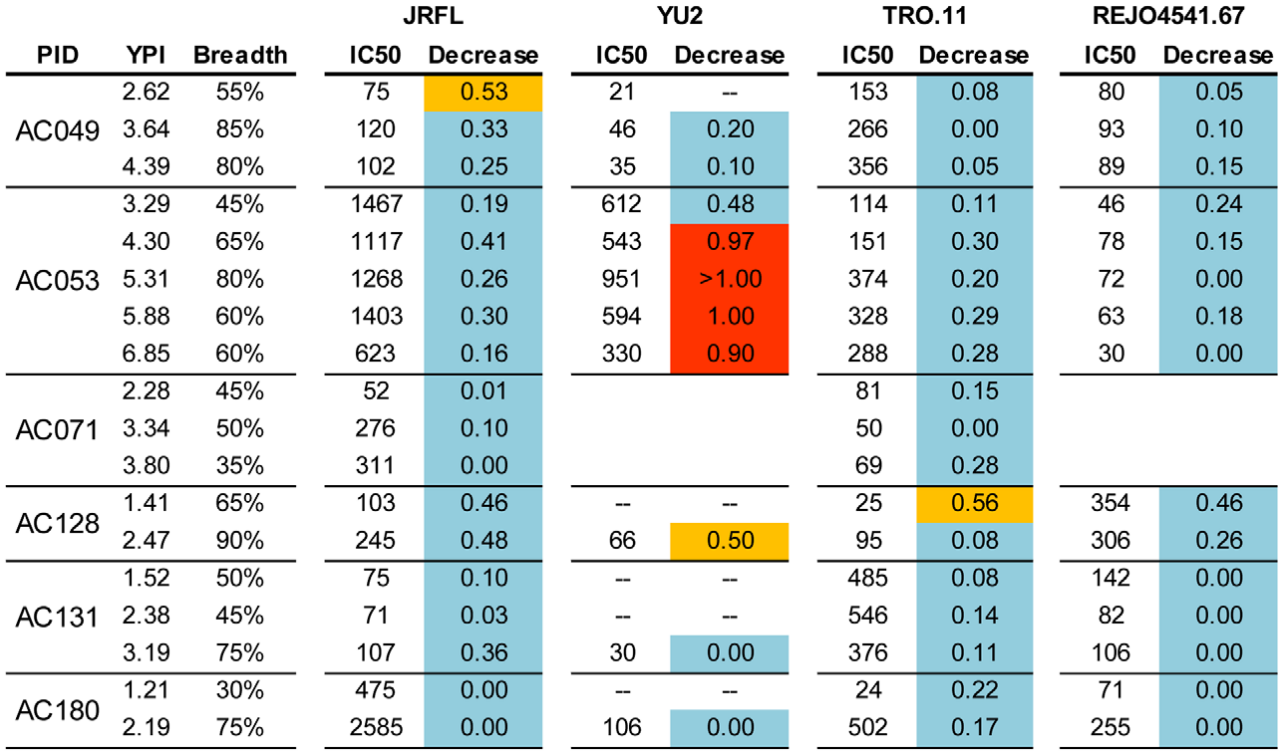
Contribution of anti-CD4-BS antibodies in the overall neutralizing activities of plasmas collected longitudinally. The values indicate the Log10 decrease in neutralizing activity in the presence of the D368R construct. The values are the average from 2–3 independent experiments in most cases. The color-coding is the same as in Figure 5. (--) the experiment was not performed because that particular plasma did not neutralize that particular virus; YPI: years post infection. Cross-neutralizing antibodies that bind the CD4-BS do not recognize the D368R mutant [19], thus, the majority of the neutralizing activities that remain in the plasmas that have been incubated with D368R most likely are due to cross-neutralizing antibodies that bind the CD4-BS. There are some exceptions, which are discussed in the Results section.

With the above caveats in mind, we examined whether the cross-neutralizing activities of plasmas that target epitopes on the D368R protein evolve over periods of time during which the breadth of cross-neutralization increases. We thus performed D368R competition neutralization experiments with plasmas collected longitudinally from subjects who gradually developed breadth (Figure 7). In the vast majority of cases, the neutralizing activities of plasmas remained minimally affected by D368R over several years of observation, as the breadth of cross-neutralization increased. The exception was plasma AC053 and virus YU2. Here, the presence of D368R had a modest (less than a 0.5 Log10 decrease in IC50) on the anti-YU2 neutralizing activity at 3.29 years following infection when breadth was less than 50%, but in the subsequent years, when breadth varied between 60% and 80%, the anti-YU2 neutralizing activity was significantly reduced (∼1Log10 or more decrease in IC50). In this particular case, therefore, the anti-YU2 neutralizing activity of this plasma gradually focused on epitopes present on D368R. This ‘focusing’ was however only observed for YU2 and not for other viruses tested, such as JRFL, TRO.11, or REJO.

## Discussion

It is now recognized that 10–30% of HIV-1 chronically infected subjects develop cross-neutralizing antibody responses of significant breadth [6], [7], [8], [12]. We and others have previously discussed that the duration of HIV infection is positively associated with the breadth of cross-neutralizing antibody responses [6], [7], [11], [12]. Here we show that such anti-viral responses become detectable in the blood of these subjects, on average, at 2.5 years after infection. In rare cases, cross-neutralizing antibodies appear as early as 1 year post-infection. A recent study indicated that the development of cross-neutralizing antibody responses does not delay the onset of AIDS [43], however, it is currently unknown whether an unusually early emergence of such responses will offer a long-term clinical benefit to the patient or not. The observation that the development of cross-neutralizing antibody responses is associated with higher levels of plasma viremia potentially indicates a more efficient viral escape from autologous anti-viral responses (neutralizing antibody responses and/or cellular-mediated anti-viral responses) in those subjects that eventually develop cross-neutralizing antibodies. Our data indicate that those subjects who develop cross-neutralizing antibodies have higher frequencies of CD4+ T expressing PD1. This observation is intriguing and potentially of high importance. A fraction of CD4+ T cells that express high levels of PD1 (termed follicular T helper cells, T_FH_) have a distinct gene expression profile from other effector T cells and develop independently of the classic TH1 or TH2 lineages [44], [45] They are not ‘exhausted’, they secrete IL-4 and IL-21 for extended periods of time, and they are crucial for the formation of germinal centers and the proliferation and survival of circulating plasma cells [46]. One possible reason for the development of broad neutralizing antibody responses in only a subset of HIV-1-infected subjects is that optimal interactions between the T_FH_ and B cells are taking place in those subjects who develop such antibody responses, while the T_FH_-B cell interactions are limited by the smaller number of T_FH_ cells in those subjects who do not develop such responses. Clearly, follow up studies are required to determine whether the CD4+PD1+ T cells found in the periphery actually behave like T_FH_ cells and whether or not the early development of cross-neutralizing antibody activities impacts the rate of disease progression.

The subjects examined here were infected with clade B HIV-1 viruses and it is not known whether the timeline for the emergence of cross-neutralizing antibody responses in non-clade B HIV-1 infections is similar, or whether infection with a particular HIV-1 subtype elicits an earlier or a delayed development of cross-neutralizing antibody responses. In the ‘early’ cases examined here, 29% of subjects developed cross-neutralizing antibody responses. This percentage is in agreement with several previous studies conducted with sera collected during chronic infection [6], [7], [8]. Potentially our data, in combination with data on the frequency of broad cross-neutralizing antibody responses in sera collected during chronic HIV-1 infection [7], [8], [47], suggest that if cross-neutralizing antibody responses are not generated during the first 2–3 years of infection, they may not emerge later. However, further follow up of these subjects is required to address this important point. It is currently unknown whether the emergence of cross-neutralizing antibody responses, at that particular time period of HIV-1 infection, in only approximately a third of those infected, is the result of a stochastic event or due to genetic predisposition, and whether it is related to particular evolutionary pathways the virus follows in response to other types of anti-viral immune responses.

In agreement with publications with sera from chronic HIV-1 infection [13], [22], we found that the earliest cross-neutralizing antibody response targets only a few regions of Env. It appears, therefore, that a few Env regions are targeted early and late during HIV-1 infection by cross-neutralizing antibodies. The fine specificities of such antibodies within these Env regions may evolve over time.

A significant portion of, or the entire, ‘early’ cross-neutralizing antibody response was due to antibodies that target virion-associated Env, rather than epitopes present on monomeric gp120 or gp41. The fact that the antibodies that bind such complex epitopes were elicited in response to infection by viruses unrelated to the heterologous viruses used to assess the cross-neutralizing potentials of HIV-1+ plasmas is strongly suggestive that these epitopes are common and most likely present on the viruses circulating even in those subjects who do not develop such antibody responses.

Since such epitopes are conserved among diverse viruses, we assume that they are also present on the transmitted viruses. Then why is the appearance of antibodies that target these epitopes delayed by 2–3 years? Potentially, antibodies that recognize epitopes which are exclusively present on the virus but not the monomeric form of HIV-1 Env could very well be generated earlier following infection, but may specifically target the autologous virus. In fact, MAbs with such complex epitope specificities that display only autologous virus neutralizing activities, or activities only against SF162 and related viruses, have been isolated from SHIV-infected macaques and from a chronically HIV-1-infected human [48], [49]. As infection progresses and in response to a continuous viral evolution, the B cell response to such complex epitopes may also evolve, and this evolution may eventually lead to the generation of antibodies with broader cross-neutralizing activities [22], [36].

The fact that anti-CD4-BS antibodies contribute to the initial cross-neutralizing activities of diverse HIV-1+ plasmas is not surprising since the CD4-BS is one of the most conserved regions of the HIV-1 Env. Numerous studies have already reported the contribution of such antibodies in defining the cross-neutralizing activities of plasmas collected during chronic HIV-1 infection [7], [13], [14], [15], [18], [19]. However, anti-CD4-BS antibodies are present in plasmas with and without ‘breadth’, and several anti-CD4-BS MAbs displaying very narrow cross-neutralizing activities have been isolated from HIV-1-infected individuals [50], [51]. Presently, it is not known why only a subset of HIV-1-infected subjects generates anti-CD4-BS antibodies that are cross-neutralizing, while the majority of subjects generate anti-CD4-BS antibodies of narrow neutralizing breadth. The angle of recognition of the CD4-BS by anti-CD4-BS antibodies with narrow and broad neutralizing activities is different [52], which implies that the CD4-BS is recognized differently by the B cell receptors (BCRs) of subjects who develop cross-neutralizing anti-CD4-BS antibodies and the BCRs of subjects who develop anti-CD4-BS antibodies of narrow neutralizing activities.

It is important to note that a fraction of the cross-neutralizing activities in some subjects could be adsorbed on both gp120 and the D368R mutant. Such specificities may be similar to those reported by Scheid et al [16] and more recently by Pietzsch et al [53] that target the ‘core’ part of gp120. Overall therefore, our data indicate that the ‘earliest’ cross-neutralizing antibody response to HIV is primarily comprised of antibodies that target the CD4-BS, the core of gp120, and epitopes present on the trimeric Env. The positive association, however, between plasma viremia levels and the breadth of the earliest cross-neutralizing antibody responses suggests that HIV is able to escape the action of the antibodies that recognize conserved regions of Env. Viral escape from antibodies that preferentially bind the Env trimeric spike may involve changes in the V1V2 region of Env, since the epitopes of this type of antibodies include elements of the V1V2 Env region [22], [36]. In fact, the V1V2 region of Env undergoes extensive alterations (including increases in length and in glycosylation) early following infection [54], [55], [56]. These changes are associated with early escape from autologous neutralizing antibody responses. Our data suggest that such changes may also be involved in the escape from the early cross-neutralizing antibody responses.

Our results provide information that may guide the development of effective immunization protocols. Since antibodies to complex epitopes that are present on the virion-associated envelope spike appear to be key components of the earliest cross-neutralizing activities of HIV-1+ plasmas, then emphasis should be made to elicit similar antibodies by vaccination. As a first step, HIV envelope glycoproteins that readily display such complex epitopes must be identified and tested as immunogens. However, if the development of such cross-neutralizing antibodies is somehow linked to genetic factors, then the outcome of immunizations with such immunogens will largely depend on the population the immunogens are evaluated, since only those vaccinees with the appropriate genetic makeup will respond appropriately.

## Materials and Methods

### Cohorts

Patients from the Vanderbilt University and the Ragon Institute of Massachusetts General Hospital ‘acute / early’ HIV infection cohorts (also referred to ‘primary’ cohorts) were used in this study. The subjects selected for the present study were infected with clade B HIV-1, had no AIDS-defining illnesses, and were not on antiretroviral therapy at the time of sample collection. In the MGH Acute HIV Infection Cohort, ‘primary infection’ was defined by detectable HIV RNA in the presence of either (i) a negative p24 ELISA, or (ii) a positive ELISA but evolving WB, or (iii) documented negative HIV ELISA within past 6 months. The plasma samples from the ‘Vanderbilt Cohort’ were collected mostly during the first year of seroconversion. All early infection subjects in this cohort had a documented negative HIV antibody test within one year of their first positive western blot result. In the case of the ‘MGH Acute HIV Infection Cohort’ the date of infection was known and samples were collected longitudinally from a few months post infection to up to 7 years post infection. In total, 53 plasma samples (collected longitudinally up to 2.5 years post-infection) from 21 HIV+ subjects from the ‘Vanderbilt Cohort’ and 69 plasma samples from 17 HIV+ subjects from the ‘MGH Cohort’ were evaluated.

### Ethics statement

The Ragon Institute's and Vanderbilt University's Institutional review boards approved the study. Written informed consent was provided by all study participants and/or their legal guardians. The data were analyzed anonymously.

### Plasma antibody adsorptions to monomeric gp120 or gp41

Plasma anti-HIV Env antibodies were adsorbed on beads coated with either recombinant SF162 gp120 or HxB2 gp41 (amino acids 541–682, Viral Therapeutics, Inc Ithaca NY) as previously described [7], [19]. The proteins were coupled to MyOne Dynabeads Tosylactivated (Invitrogen) following the manufacturer's instructions. Briefly, 40 mg of magnetic beads were reacted with 1 mg protein ligand overnight at 37°C with gentle rotation. After collecting the beads on a magnet, the supernatant was removed and the beads were incubated overnight at 37°C in PBS, 0.5% BSA, 0.05% Tween 20. The magnetic beads were washed twice with PBS, 0.1% BSA, 0.05% Tween 20, and stored at 4°C in the same buffer, with the addition of 0.02% Sodium Azide. Bead-coupled Env proteins were tested for antigenic integrity by flow cytometry using known MAbs b12, 447–52D, 2G12, IgG-CD4, and 4E10, followed by detection with goat-anti-human-IgG-FITC secondary antibody (data not shown). Mock adsorption/elution experiments using several anti-HIV Env MAbs at a concentration of 10 µg/ml in naïve plasma were performed as a positive control (data not shown). 500µl of plasma, diluted 1∶5 in DMEM/10%FBS, were incubated with 200µl Env protein-coupled beads at room temperature for 120 min with gentle rotation. The samples were placed on a magnet and the beads were isolated.

The antibodies bound to the bead-coupled Env proteins were eluted in a series of increasingly acidic solutions as previously described [19]. The beads from each serial adsorption were combined and incubated in 0.1M Glycine-HCl, pH 2.7 for 30 seconds with vortexing. The beads were collected by brief centrifugation and held in place by a magnet. The supernatant was removed and adjusted to pH 7.5 with 1M Tris (pH 9.0). The process was repeated with the beads in 0.1M Glycine-HCl, pH 2.3 and then again in pH 1.7. The final supernatants were buffer-exchanged in PBS and washed over a 30kD Amicon Ultra centrifugation concentrator (Millipore). Concentration of immunoglobulin was determined by absorbance at 280 nm (NanoDrop Spectrophotometer ND-1000, Thermo). The depleted plasmas and the antibodies that were eluted from gp120-coated beads were tested by ELISA for reactivity to gp120, and for neutralizing activity.

### Neutralization assays

The neutralizing activities of plasmas were determined using the Tzm-bl-based neutralization assay [57]. Briefly, plasma dilutions (starting at 1∶20) were pre-incubated with single-round competent virions (pseudovirus) for 60 minutes at 37°C. The plasma / pseudovirus mixture was added to TZM-bl cells (3000 cells per well in a 96-well plate) for 72 hrs at 37°C. The supernatant was removed and 100µl of Steady-Glo Luciferase Assay Substrate (Promega) was added to each well. Plates were incubated for 15 minutes at room temperature and 75µl of the lysate was transferred to micro titer plates. The cell-associated luciferase activity for each well was determined on a Fluoroscan Luminometer (Thermo). Percent neutralization was calculated at each plasma dilution as the percent inhibition of viral entry by the plasma sample compared to the absence of plasma. For each plasma/virus combination tested, a neutralization curve (percent neutralization versus plasma dilution) was generated using GraphPad Prism version 4.03 for Windows (GraphPad Software, San Diego California, USA), and the plasma dilution at which 50% neutralization was recorded (IC50) was determined by transforming the data to a log10 scale with fitted sigmoidal dose-response curves.

Neutralization breadth of a plasma sample is defined as the percent (0%–100%) of the 20 isolates neutralized by that sample.

All plasmas were tested against single round competent virions expressing Envs from 10 Clade B, 6 Clade C and 4 Clade A primary viruses. The clade B SF162.LS (EU123924), JRFL (U63632) and YU2 (M93258) viruses were isolated during chronic HIV-1 infection and the remaining isolates were isolated during acute infection, with published accession numbers [58], [59], [60], [61]. All plasma samples were also screened for non-HIV-specific neutralization using the murine leukemia virus (MLV) pseudotyped into the HIV backbone. Neutralization activity was not detected against MLV at 1∶20 by any of the plasma samples (data not shown).

In certain cases, competition neutralization experiments were performed in the presence of the D368R gp120 or an MPER-derived peptide. Serially diluted MAbs or HIV+ plasmas were pre-incubated with D368R (25 µg/ml) or the MPER peptide (10 µg/ml) for 1 hour at 37°C and then the mixture was incubated with virus for another hour at 37°C, and subsequently with cells as described above. The fold decrease in log10 IC50 neutralization titers of each plasma tested against each virus in the presence of D368R or the MPER peptide was determined.

### Statistical analysis

Logarithmic transformation was used for viral load, and nonparametric regression with two-tailed p-value analysis was used to determine correlations between the breadth of cross-neutralizing antibody responses in HIV-1+ plasmas and plasma viremia levels. Mann-Whitney Test and Pierson correlation and linear regression analysis were used to determine correlations between immune activation and breadth of neutralizing activities.

## Supporting Information

Figure S1 Depletion of anti-gp120 antibodies. The indicated plasmas were depleted from their anti-gp120 antibodies, and their anti-gp120 reactivities prior to and following depletion are shown. The anti-gp120 reactivities were evaluated against 4 heterologous gp120s: (A) SF162.LS, (B) JRFL, (C) QH069.42, and (D) Du422.1. Black lines: undepleted plasmas; blue lines: gp120-depleted plasmas. Years post infection (time following infection he plasmas were collected) and breadth are indicated in parenthesis in (A) next to each subjects ID.
(0.56 MB TIF)

Figure S2 Effect of N160 mutation on the neutralizing activities of MAbs PG9 and PG16. The neutralizing activities of MAbs (A) PG9 and (B) PG16 against TRO.11 are shown. WT: wild type TRO.11; N160K: TRO.11 with the asparagine at position 160 mutated to a lysine; N160A: TRO.11 with the asparagine at position 160 mutated to an alanine.
(0.19 MB TIF)

Figure S3 Competing the neutralizing activities of known MAbs by D368R. The neutralizing activities of known anti-HIV neutralizing MAbs were determined in the presence and absence of the competing D368R gp120 protein. (A) Neutralizing activities of the anti-V1 MAb P3C8, anti-V3 MAbs P3E1 and 447D, and MAb 2G12 (recognizes a complex glycan epitope on gp120). (B) Neutralizing activities of the anti-CD4-BS MAb b12 and of IgGCD4 are shown. Solid lines and symbols: absence of D368R; dashed lines and open symbols: presence of D368R.
(0.28 MB TIF)

Figure S4 Neutralizing activities of HIV+ plasmas in the presence of the D368R mutant gp120. The neutralizing activities of plasmas (A) AC049, (B) AC053, and (C) AC180 against TRO.11 (red squares), JRFL (blue triangles) and YU2 (green circles) were determined in the absence (solid lines and symbols) and presence (dotted lines and open symbols) of D368R gp120. Patient ID, breadth, and years post infection are shown.
(0.30 MB TIF)
