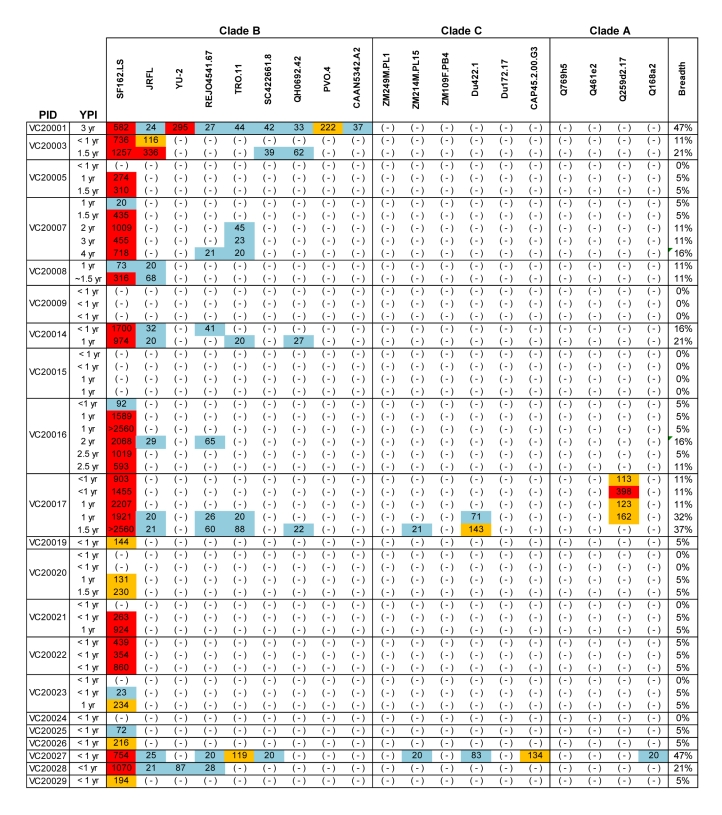# Correction: Characteristics of the Earliest Cross-Neutralizing Antibody Response to HIV-1

**DOI:** 10.1371/annotation/8b3b24b5-d4ed-483a-b233-0a88513ad499

**Published:** 2011-03-01

**Authors:** Iliyana Mikell, D. Noah Sather, Spyros A. Kalams, Marcus Altfeld, Galit Alter, Leonidas Stamatatos

Figure 1 is incorrect. Please see the corrected Figure 1 here: 

**Figure:**