# Supplementary figures and images for: Characteristics of the Earliest Cross-Neutralizing Antibody Response to HIV-1
Source: PLoS Pathog. 2011 Jan 13;7(1):e1001251. doi: 10.1371/journal.ppat.1001251 (PMC3020924; doi:10.1371/journal.ppat.1001251)

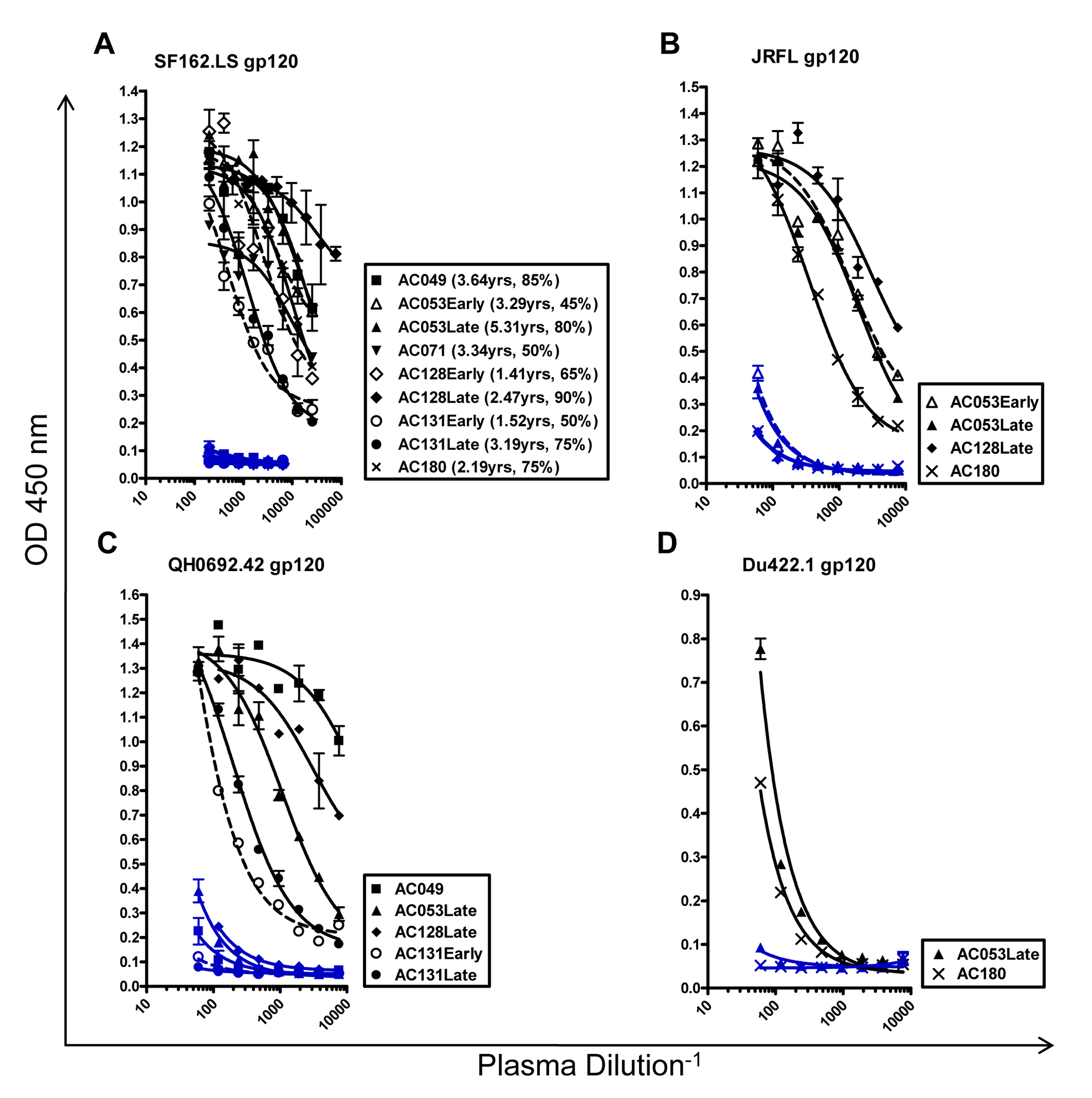

Supplement: Figure S1 — Depletion of anti-gp120 antibodies. The indicated plasmas were depleted from their anti-gp120 antibodies, and their anti-gp120 reactivities prior to and following depletion are shown. The anti-gp120 reactivities were evaluated against 4 heterologous gp120s: (A) SF162.LS, (B) JRFL, (C) QH069.42, and (D) Du422.1. Black lines: undepleted plasmas; blue lines: gp120-depleted plasmas. Years post infection (time following infection he plasmas were collected) and breadth are indicated in parenthesis in (A) next to each subjects ID. (0.56 MB TIF) [file ppat.1001251.s001.tif]

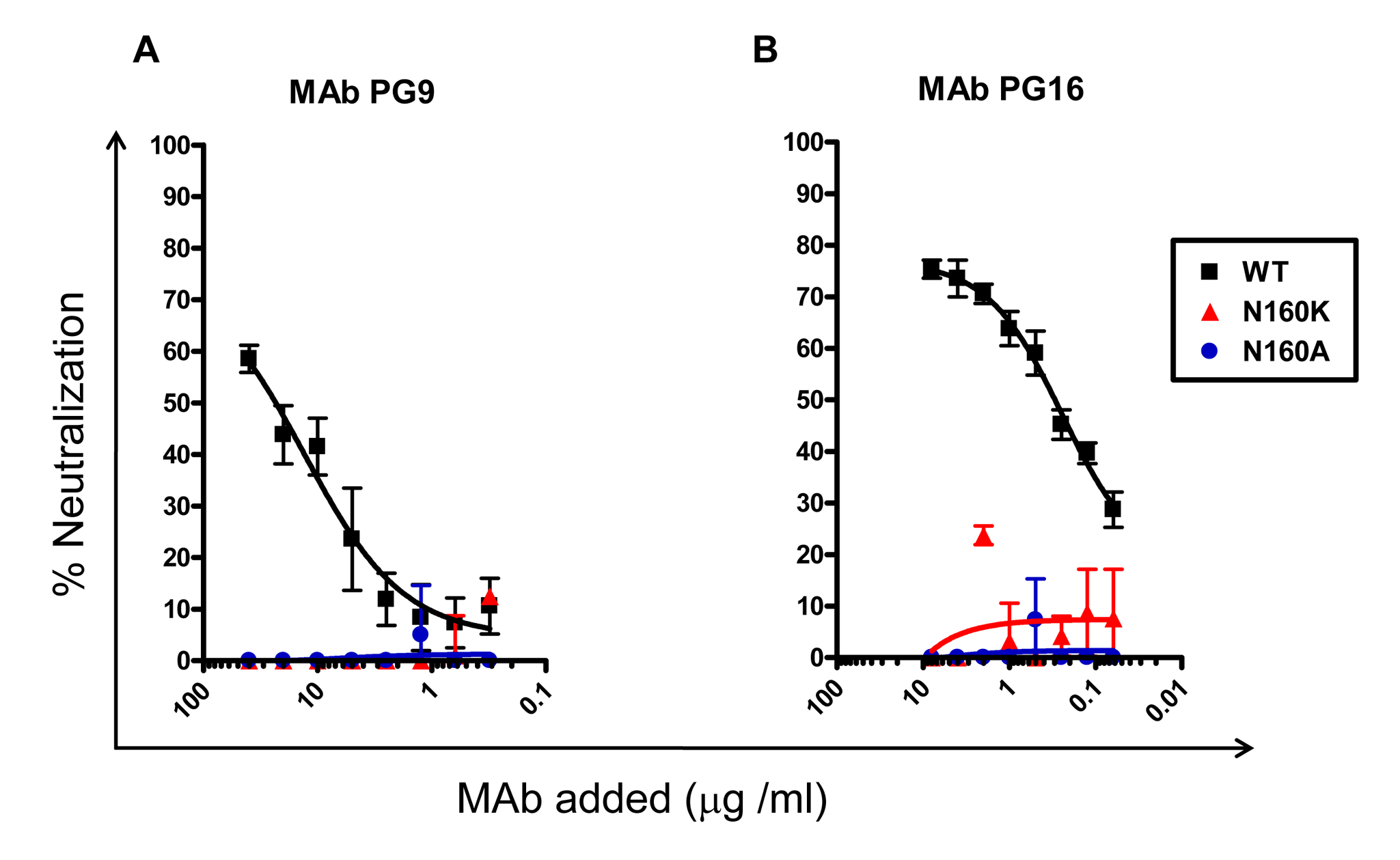

Supplement: Figure S2 — Effect of N160 mutation on the neutralizing activities of MAbs PG9 and PG16. The neutralizing activities of MAbs (A) PG9 and (B) PG16 against TRO.11 are shown. WT: wild type TRO.11; N160K: TRO.11 with the asparagine at position 160 mutated to a lysine; N160A: TRO.11 with the asparagine at position 160 mutated to an alanine. (0.19 MB TIF) [file ppat.1001251.s002.tif]

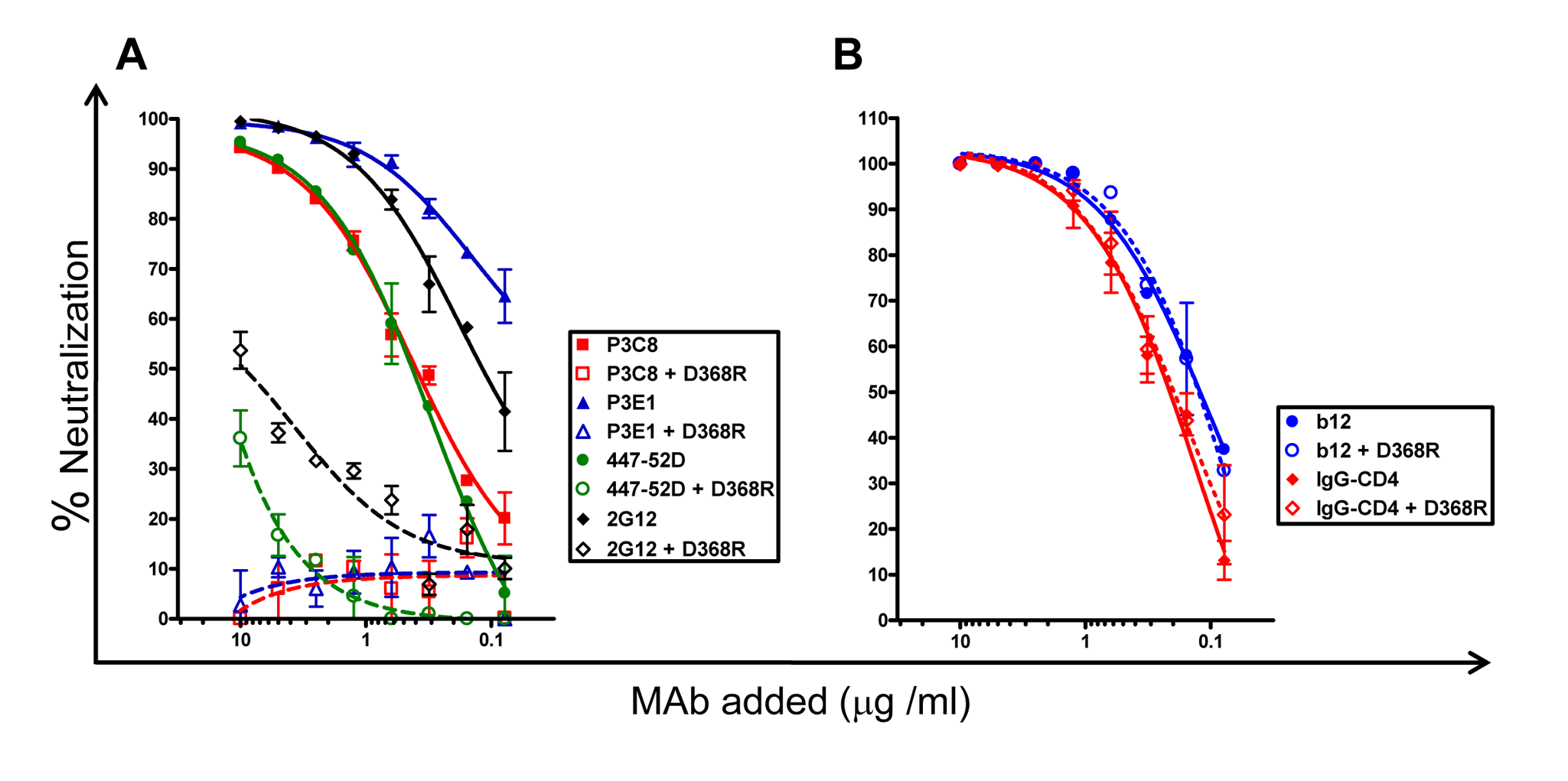

Supplement: Figure S3 — Competing the neutralizing activities of known MAbs by D368R. The neutralizing activities of known anti-HIV neutralizing MAbs were determined in the presence and absence of the competing D368R gp120 protein. (A) Neutralizing activities of the anti-V1 MAb P3C8, anti-V3 MAbs P3E1 and 447D, and MAb 2G12 (recognizes a complex glycan epitope on gp120). (B) Neutralizing activities of the anti-CD4-BS MAb b12 and of IgGCD4 are shown. Solid lines and symbols: absence of D368R; dashed lines and open symbols: presence of D368R. (0.28 MB TIF) [file ppat.1001251.s003.tif]

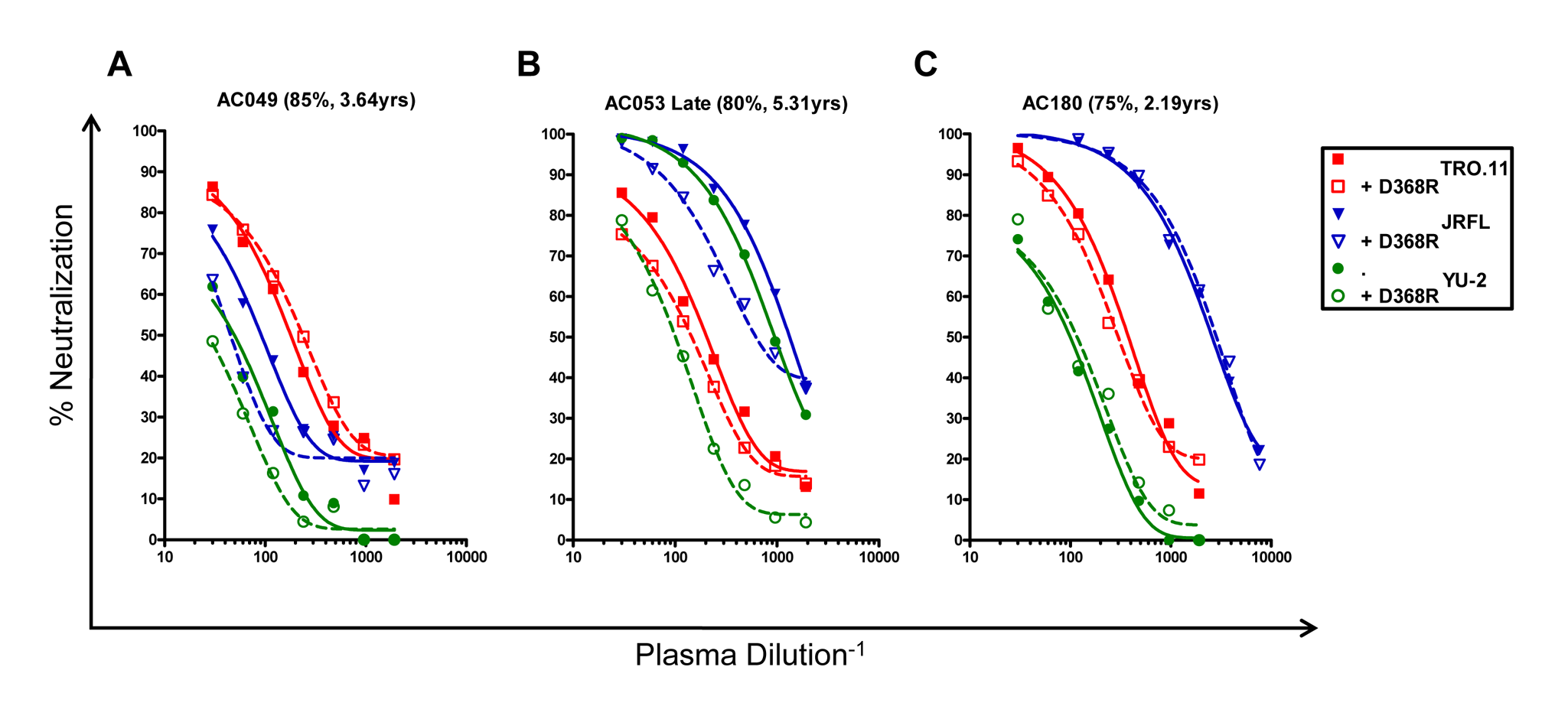

Supplement: Figure S4 — Neutralizing activities of HIV+ plasmas in the presence of the D368R mutant gp120. The neutralizing activities of plasmas (A) AC049, (B) AC053, and (C) AC180 against TRO.11 (red squares), JRFL (blue triangles) and YU2 (green circles) were determined in the absence (solid lines and symbols) and presence (dotted lines and open symbols) of D368R gp120. Patient ID, breadth, and years post infection are shown. (0.30 MB TIF) [file ppat.1001251.s004.tif]
